# Tirzepatide reduced alcohol use in three patients with alcohol-associated liver disease, type 2 diabetes, and obesity

**DOI:** 10.1530/EDM-26-0002

**Published:** 2026-04-01

**Authors:** Hiroki Takizawa, Akiko Tsuda, Hidehisa Suriki, Hideki Amagane

**Affiliations:** ^1^ Department of Internal Medicine, Kido Hospital, Niigata, Japan; ^2^ Department of Internal Medicine, Niigata University Faculty of Medicine, Niigata, Japan; ^3^ Department of Psychiatry, Kohdo Hospital, Niigata, Japan

**Keywords:** diabetes, obesity, alcohol-associated liver disease, tirzepatide, alcohol use disorder

## Abstract

**Summary:**

We report three obese patients with type 2 diabetes and alcohol-associated liver disease (ALD), including one patient with alcohol use disorder, who exhibited reduced alcohol intake after the initiation of tirzepatide. Although tirzepatide has shown beneficial effects on liver fibrosis in metabolic dysfunction-associated steatohepatitis, its effect on ALD or alcohol intake has not been reported. After the initiation of tirzepatide, all patients demonstrated decreased alcohol consumption, improved liver function, and better metabolic control. These findings suggest that tirzepatide can provide additional benefits for ALD by reducing alcohol intake while improving metabolic risk factors, potentially offering a novel therapeutic option for this high-risk population.

**Learning points:**

- The interaction between alcohol consumption and metabolic risk factors in liver disease is bidirectional, necessitating concurrent improvement of both aspects.
- Treatment with tirzepatide for type 2 diabetes was associated with reduced alcohol consumption in our cases.
- Tirzepatide might represent an optimal therapeutic option for patients with alcohol-associated liver disease and type 2 diabetes given its potential to reduce alcohol consumption and ameliorate metabolic risk factors.

## Background

Alcohol is the major cause of preventable liver morbidity and mortality globally. Alcohol-associated liver disease (ALD) encompasses a wide range of liver disorders that can eventually lead to cirrhosis. Consequently, ALD is the most common cause of cirrhosis. Despite the critical importance of reducing alcohol intake, including abstinence, to prevent cirrhosis and liver cancer in patients with ALD, there are no approved targeted therapies (1). ALD confers a particularly high risk of cirrhosis in the presence of metabolic risk factors, such as obesity and diabetes (2). Thus, in such patients, treatment of metabolic risk factors is also necessary.

Tirzepatide, a dual agonist of the glucagon-like peptide-1 (GLP-1) and glucose-dependent insulinotropic polypeptide (GIP) receptors, has been reported to improve liver fibrosis in patients with metabolic dysfunction-associated steatohepatitis (MASH) (3), although patients with ALD were excluded from the trial. Furthermore, the effects of tirzepatide on alcohol intake and alcohol use disorder (AUD) have not been reported.

Here, we report a decrease in alcohol consumption and improvement in metabolic risk factors and liver function after treatment with tirzepatide in patients with obesity, type 2 diabetes, and ALD.

## Case presentation

### Case 1

The patient was a 49-year-old man with dyslipidemia, hypertension, and hyperuricemia. His family history included a paternal uncle with type 2 diabetes. At 43 years old, he was diagnosed with AUD and ALD. His ethanol consumption ranged from 107 to 284 g/day since his twenties. At 46, he was dismissed from his job after testing positive for alcohol at work. Treatment with nalmefene reduced his ethanol intake to 40 g/day.

### Case 2

The patient was a 66-year-old man with hypertension. His ethanol consumption ranged from 413 to 553 g/week from age 20.

### Case 3

The patient was a 52-year-old woman with dyslipidemia, hypertension, bronchial asthma, and adjustment disorder. Her family history included a father with type 2 diabetes. At 51 years old, she was diagnosed with diabetes (height: 159 cm; weight: 73 kg; body mass index (BMI): 28.9 kg/m^2^), and she initiated metformin and empagliflozin therapy. Her ethanol intake started at age 20 and ranged from 276 to 620 g/week, but it decreased to 42–84 g/week after the first visit.

## Investigation

### Case 1

Serological tests were negative, and liver biopsy confirmed ALD (METAVIR A2F2). Despite a history of falling and breaking bones while intoxicated and hospitalization for vomiting related to alcohol consumption, he continued to consume 55 g of ethanol/day, and his liver enzyme levels remained elevated (aspartate aminotransferase (AST): 60–120 U/L (reference range (RR): 13–33 U/L); alanine aminotransferase (ALT): 100–180 U/L (RR for men: 8–42 U/L); gamma-glutamyl transferase (GGT): 1,000–2,000 U/L (RR: 10–47 U/L)). A subsequent health examination revealed hyperglycemia (height: 166 cm; weight: 91.4 kg; BMI: 33.2 kg/m^2^; AST: 85 U/L; ALT: 86 U/L; GGT: 904 U/L; FIB-4 index: 3.33; hemoglobin A1c (HbA1c): 8.8%; glucose: 182 mg/dL (10.1 mmol/L)).

### Case 2

At 63 years old, he presented with the following findings – HbA1c: 6.0%; fasting glucose: 136 mg/dL (7.55 mmol/L); height: 169 cm; weight: 95 kg (BMI: 33.3 kg/m^2^); and fatty liver on ultrasound. At 65, his liver and metabolic indices worsened (AST: 117 U/L; ALT: 183 U/L; GGT: 220 U/L; FIB-4 index: 2.26; HbA1c: 7.2%; weight: 99.4 kg), and serological tests were negative.

### Case 3

Ultrasound revealed fatty liver. At 52, her liver and glucose parameters worsened (AST: 73 U/L; ALT: 69 U/L (RR for women: 6–27 U/L); GGT: 19 U/L; FIB-4 index: 1.18; HbA1c: 6.5%; weight: 74.5 kg).

## Treatment

### Case 1

Subcutaneous tirzepatide at 2.5 mg/week was initiated.

### Case 2

Subcutaneous tirzepatide at 2.5 mg/week was initiated, and the dose was increased to 5 mg/week after 4 weeks.

### Case 3

Subcutaneous tirzepatide at 2.5 mg/week was initiated. Due to nausea lasting 3 days after each injection, dose escalation was postponed. However, as the symptoms gradually improved, the dose was increased to 5 mg/week after 11 weeks. No other antidiabetic medications were changed.

The dose of tirzepatide was maintained at 2.5 mg/week in case 1 and 5 mg/week in cases 2 and 3, based on patient preferences and the achievement of adequate glycemic control.

## Outcome and follow-up

### Case 1

One month after the initiation of tirzepatide, the patient’s ethanol intake decreased to 28 g/day, and his alcohol cravings declined. The dose of tirzepatide was not increased due to transient postprandial lethargy. After 5 months, his FIB-4 index, HbA1c, and body weight decreased to 2.57, 5.7%, and 85.3 kg, respectively.

### Case 2

Eight months after the initiation of tirzepatide, his liver function and metabolic indices improved (AST: 32 U/L; ALT: 48 U/L; GGT: 59 U/L; FIB-4 index: 1.12; HbA1c: 5.8%; weight: 96.3 kg). His ethanol intake decreased to 56–78 g/week, and he noted that he ‘became gradually bored while drinking’.

### Case 3

Six months after the initiation of tirzepatide, her liver function and metabolic indices improved (AST: 22 U/L; ALT: 20 U/L; GGT: 8 U/L; FIB-4 index: 0.64; HbA1c: 5.5%; weight: 65.1 kg), and her ethanol intake decreased to 14 g/month (3 g/week).

In summary, we present three patients with type 2 diabetes and ALD whose alcohol intake markedly decreased from one-half to one-twentieth after the initiation of tirzepatide without additional encouragement or intentional efforts to reduce alcohol consumption.

## Discussion

The interaction between alcohol and metabolic risk factors such as hyperglycemia and obesity in steatotic liver disease is bidirectional (1, 2, 4), indicating the need to improve all parameters simultaneously. Recently, GLP-1 receptor agonists, particularly semaglutide, have been reported to decrease alcohol intake in AUD (5). This observation may reflect modulation of reward-related pathways involved in alcohol craving, which have been suggested to be influenced by central GLP-1 signaling.

Tirzepatide, a dual GLP-1/GIP agonist, has favorable effects on steatotic liver disease and metabolic risk factors, including obesity. The drug has been shown to improve liver fibrosis in patients with metabolic dysfunction-associated steatohepatitis (3), although patients with ALD were excluded. Furthermore, tirzepatide induces greater weight loss than semaglutide (6). All three patients in our series displayed improvements in metabolic risk factors, accompanied by improvements in liver function. GIP receptor agonism might also modulate appetite through hypothalamic receptors (7), but its potential role in suppressing alcohol consumption remains unclear.

Importantly, the reduction in alcohol intake in our series was not explained by persistent gastrointestinal adverse effects that might have mechanically limited drinking behavior. One patient reported that he ‘became gradually bored while drinking’, which may suggest reduced reward from alcohol.

There are barriers for both the public and healthcare providers in recognizing and treating ALD and AUD (1). The belief that abstinence is the only therapeutic strategy could discourage treatment (8). Recently, reduced alcohol intake has been recommended by health organizations such as the European Medicines Agency as an appropriate surrogate endpoint for improving morbidity and mortality (8). Given the limited awareness of alcohol use as a modifiable factor, it is important to note that alcohol consumption was reduced, as in the present cases, without deliberate effort or specific counseling.

Several limitations must be acknowledged. First, liver biopsies were not performed in all patients. Second, the amount of alcohol consumed was self-reported. Although these factors are sometimes acceptable in clinical settings where liver biopsy and standardized questionnaires are not readily available, a more rigorous evaluation would have been desirable. Third, because of the relatively low dose of tirzepatide, it remains unclear whether dose escalation would further reduce alcohol consumption. Therefore, the relationship between tirzepatide dose and alcohol reduction remains to be clarified in future studies. Since the effects on body weight (6) and MASH (3) have typically been evaluated at doses of 5 mg or higher, and higher doses have been shown to be more effective, the potential benefit of higher doses should also be considered.

In conclusion, tirzepatide might be a valuable therapeutic option for patients with type 2 diabetes and ALD, offering multiple benefits: reduced alcohol consumption, improved metabolic risk factors, and favorable effects on steatotic liver disease.

## Declaration of interest

There is no conflict of interest that could be perceived as prejudicing the impartiality of the research reported.

## Funding

This research did not receive any specific grant from any funding agency in the public, commercial, or not-for-profit sector.

## Patient consent

Written informed consent for publication of their clinical details was obtained from the patients.

## Author contribution statement

All authors made individual contributions to authorship. All authors were involved in the diagnosis and management of the patients. HT was involved in the manuscript submission. All authors reviewed and approved the final draft.
